# Prognostic value of C-reactive protein levels in patients with bone neoplasms: A meta-analysis

**DOI:** 10.1371/journal.pone.0195769

**Published:** 2018-04-18

**Authors:** Wenyi Li, Xujun Luo, Zhongyue Liu, Yanqiao Chen, Zhihong Li

**Affiliations:** 1 Department of Orthopedic, The Second Xiangya Hospital, Central South University, Changsha, Hunan, China; 2 Department of Cardiovascular Medicine, The Second Xiangya Hospital, Central South University, Changsha, Hunan, China; University of Nebraska Medical Center, UNITED STATES

## Abstract

**Objective:**

The aim of this study was to conduct a meta-analysis of retrospective studies that investigated the association of preoperative C-reactive protein (CRP) levels with the overall survival (OS) of patients with bone neoplasms.

**Methods:**

A detailed literature search was performed in the Cochrane Library, Web of Science, Embase and PubMed databases up to August 28, 2017, for related research publications written in English. We extracted the data from these studies and combined the hazard ratios (HR) and 95% confidence intervals (CIs) to assess the correlation between CRP levels and OS in patients with bone neoplasms.

**Results:**

Five studies with a total of 816 participants from several countries were enrolled in this current meta-analysis. In a pooled analysis of all the publications, increased serum CRP levels had an adverse prognostic effect on the overall survival of patients with bone neoplasms. However, the combined data showed no significant relationship between the level of CRP and OS in Asian patients (HR = 1.73; 95% CI: 0.86–3.49; P = 0.125). Similar trends were observed in patients with bone neoplasms when stratified by ethnicity, histology, metastasis and study sample size.

**Conclusions:**

The results of this meta-analysis suggest that increased CRP expression indicates a poorer prognosis in patients with bone neoplasms. More prospective studies are needed to confirm the prognostic significance of CRP levels in patients with bone neoplasms.

## Introduction

Primary neoplasms of bone, namely osteosarcoma, chondrosarcoma, and the Ewing’s sarcoma family of tumors, are estimated to affect 3240 new patients and to cause 1550 deaths each year in the U.S. [1]. Osteosarcoma and Ewing’s sarcoma occur most commonly in teenagers, while chondrosarcoma occurs more frequently in older adults. Despite advances in surgical techniques and continuous efforts to improve therapy regimens, the 5-year relative survival rates of bone cancers have remained relatively stable since the 1980s. Therefore, multiple studies are being conducted to determine new markers for prognosis and targets for prospective treatments for those cancers [2].

C-reactive protein (CRP) is a common acute phase serum protein. It was first discovered in the plasma of patients with pneumonia and was named after its reactivity with the C polysaccharide derived from the pneumococcal cell wall [3]. CRP can interact with multiple ligands and receptors, including phosphocholine (PC) on pathogenic organism and damaged cell membranes, nuclear antigens, C1q in the classical complement pathway, and FcγRI and FcγRII on the surface of leukocytes, allowing it to play an important role in innate immunity [4]. CRP is produced by hepatocytes, mainly in response to interleukin-6 (IL-6) secreted by T cells and macrophages, which regulates CRP production at the transcriptional level [5]. In the circulating blood of healthy adults, CRP is only present in trace amounts, but its level rapidly rises within 2 hours of the onset of trauma, infection, and inflammation, and decreases quickly after the resolution of such conditions. Therefore, it has been used as a common marker for inflammation. Interestingly, CRP has been proven to be strongly associated with various cancers, exhibiting diagnostic or prognostic value [6–13]. While multiple studies have been carried out, the relationship between CRP and the prognosis of bone cancer remains controversial, with end results varying among studies. To evaluate the significance of the preoperative level of CRP for the outcome of bone cancer patients, we did a systematic review and meta-analysis using updated data on individual patients from all available trials.

## Methods

### Compliance with ethical standards

Ethical approval: All procedures performed in studies involving human participants were performed in accordance with the ethical standards of the institutional and/or the national research committee and with the 1964 Helsinki declaration and its later amendment.

### Search strategy

PubMed, Cochrane Library, Web of Science and Embase were thoroughly searched up to August 28, 2017 using the following key words: “C-reactive protein”, “bone neoplasms” and “bone cancer”. All studies identified in this manner were retrieved. The references of the selected studies were also searched for other relevant studies. The publication language was limited to English. The titles and abstracts of the selected studies were screened to filter appropriate studies, and the full texts were evaluated carefully. There were no restrictions on the number of patients in these published studies. This meta-analysis was registered in PROSPERO (http://www.crd.york.ac.uk/PROSPERO), and the registration number for this article is CRD42017075200.

### Eligibility criteria

The following inclusion criteria were used: 1) patients included in studies were pathologically diagnosed as having bone neoplasms; 2) the level of CRP was evaluated before treatment; 3) the study provided HRs and 95% CIs for CRP in terms of OS or the data necessary to calculate them; 4) the publications were written in English. The exclusion criteria were as follows: 1) articles that were reviews, meeting abstracts, or letters, or lacking the full text in English; 2) nonhuman studies; 3) studies that did not provide the levels of CRP before treatment. If the data sets overlapped or were duplicated, only the most recent information was included in this meta-analysis. All identified studies were investigated independently for eligibility by two authors.

### Data extraction

Two independent authors (W, L and X, L) independently extracted information from the eligible studies, and any disagreement between them was resolved by discussion and consensus. The following information was recorded from the 5 included studies: the surname of the first author, the study country, the year of publication, the sample size, the survival data and the detailed information regarding the bone neoplasms.

### Quality assessment

The quality assessment of the primary studies was performed according to the Newcastle-Ottawa quality assessment scale (NOS). The maximum possible score is 9 points, and studies scoring ≥6 points were regarded as high-quality studies (http://www.ohri.ca/programs/clinical_epidemiology/oxford.asp).

### Statistical analysis

All statistical analysis was carried out by STATA 14.0 (STATA Corp, College Station, TX) [14]. Statistical heterogeneity among the studies was evaluated with Q and I^2^ statistics, with the significance level set at p <0.05 [15]. If there was significant heterogeneity among the studies, the random effects model was used to calculate the pooled HR and 95% CI [16]. The potential publication bias was estimated using Begg’s test [17]. P< 0.10 was considered statistically significant. Sensitivity analyses were performed by excluding each study individually from the meta-analysis.

## Result

### The characteristics of the included studies

**Fig 1:**
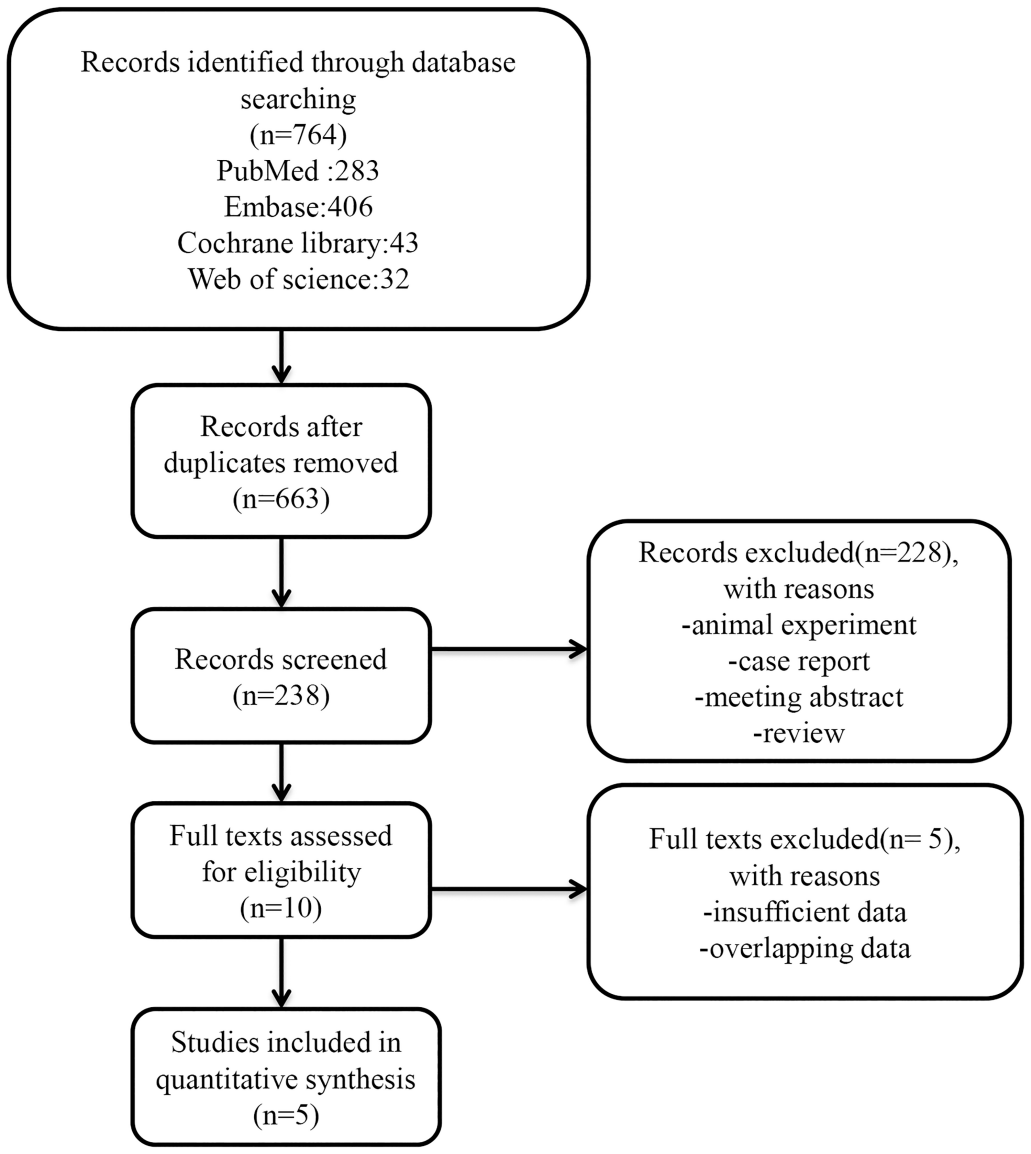
Flow chart of the study selection.

The flow diagram of the current study is presented in Fig 1. Five relevant studies with a total of 816 patients were selected for initial review by the search strategies described above [18–22]. The sample sizes ranged from 85 to 318 participants. All the enrolled studies were retrospective. Of these studies, three [18, 19, 22] were carried out in Europe, and the remaining studies were conducted in Asia. Only two publications [18, 22] involved patients with osteosarcoma and other kinds of bone neoplasms, and the other three studies focused on osteosarcoma [19–21]. An elevated level of CRP was defined as ≥8 mg/l or ≥75 nmol/l in one study [18]; otherwise, a normal level of CRP was defined as less than 10 mg/dl. Among the participants, there were 671 patients with distant metastasis, and 145 with no distant metastasis. All enrolled publications defined OS as the time from diagnosis to the day of death or the day of the last follow-up. The articles were published between 2011 and 2016, and the NOS scores of the included studies ranged from 6–9. The detailed information is shown in Table 1.

**Table 1:** Characteristics of all included studies.

Study	Year	Location	Ethnicity	Follow-up	Sample size	Gender(M/F)	Type	Treatment	Outcomes	Metastasis	NOS
Liu, B	2016	China	Asian	NA	162	96/66	OST	S,C and R	OS	78/84	7
Aggerholm-Pedersen, N.	2016	Denmark	European	8.8 year(median)	172	98/74	CH = 62 EW/OST = 109	S and C	OS/disease-specific survival	NO	8
Li, X. C.	2015	China	Asian	NA	85	43/42	OST	C	OS	37/48	6
Nakamura, T.	2013	British	European	40 month(mean)	318	176/142	OST = 112C H = 93 EW = 47 Other = 66	S,C and R	OS	NO	8
Funovics, P. T.	2011	Austria	European	46 month(mean)	79	42/37	OST	S and C	OS/disease-specific survival	30/49	8

Abbreviations: CH: chondrosarcoma; EW: Ewing’s sarcoma; OST: osteosarcoma; OS: overall survival; NOS: Newcastle-Ottawa quality assessment scale; S: surgery; C: chemotherapy; R: radiotherapy; NA: data were not provided in the publication.

### Relationship between CRP and OS in bone neoplasms

**Fig 2:**
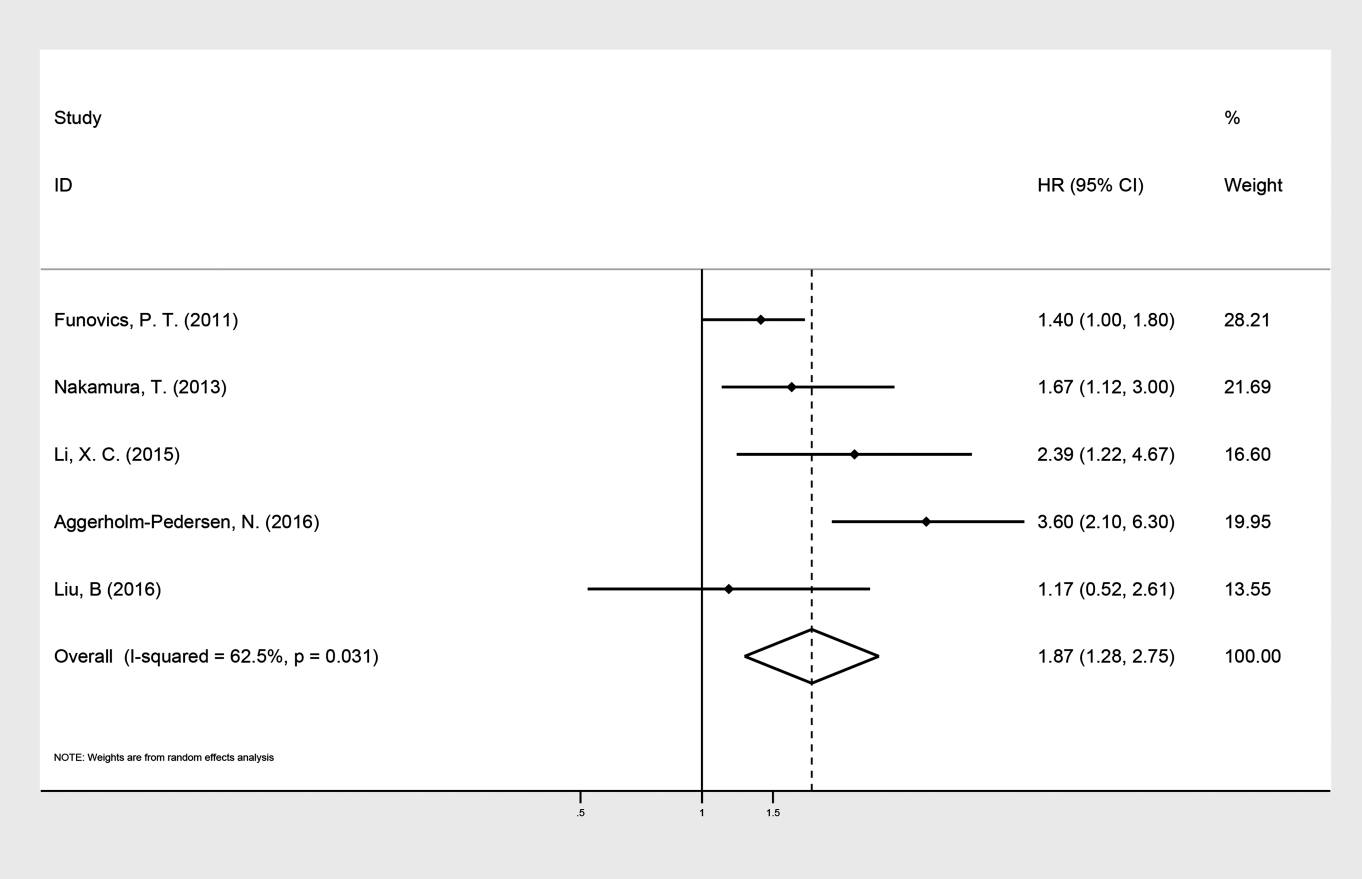
Forest plot of the association between the level of CRP and OS in patients with bone neoplasms. Summary of estimated hazard ratios (HRs) and 95% CI for patients with bone neoplasms.

The five selected studies provided the levels of CRP before treatment and OS in patients with bone neoplasms. The random effects model showed a significant relationship between elevated levels of CRP and OS in patients with bone neoplasms (HR: 1.87; 95% CI: 1.28–2.75; P = 0.001), with heterogeneity (I^2^ = 62.4%, P = 0.031, Fig 2).

### Subgroup analyses

**Fig 3:**
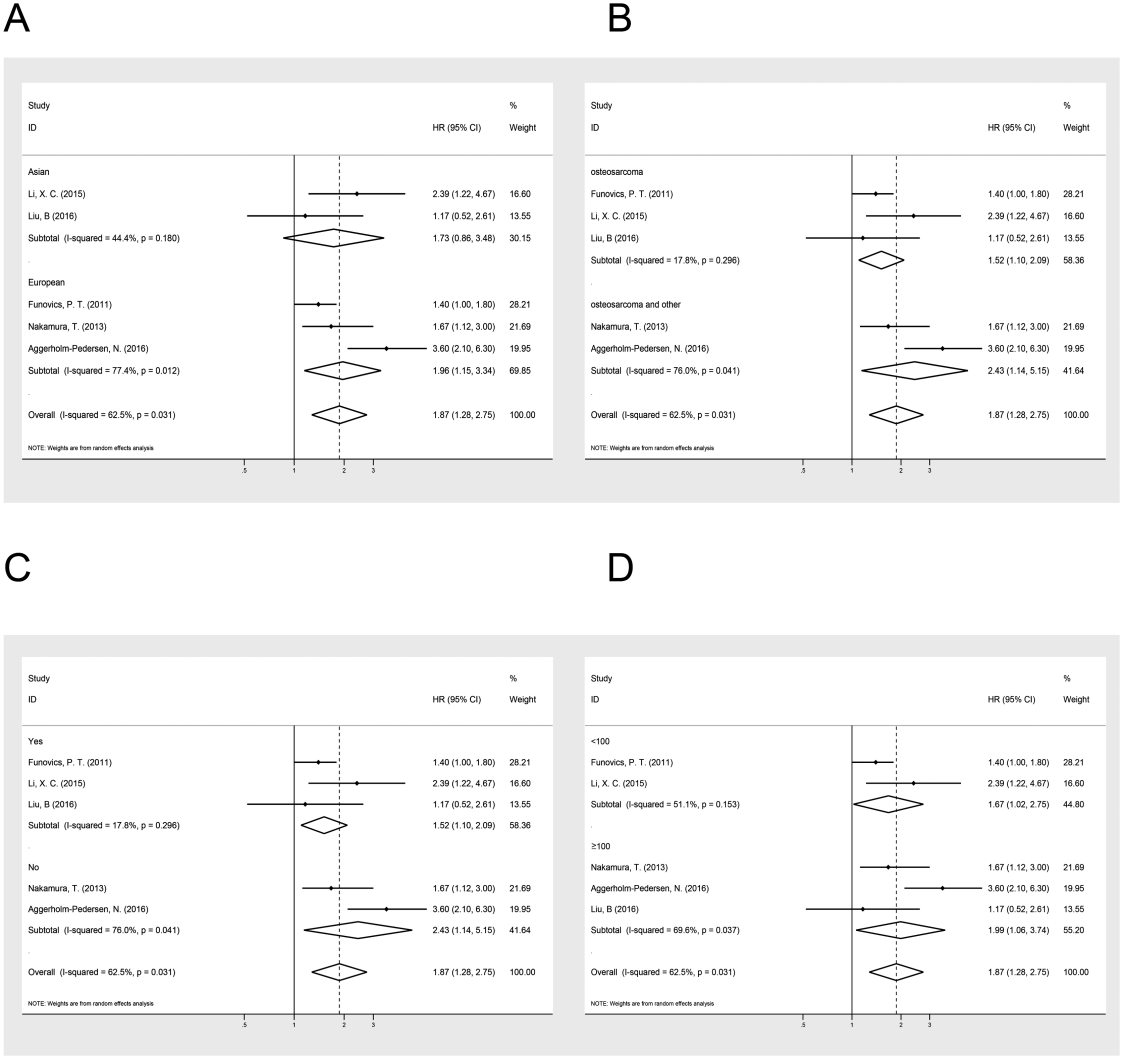
Forest plot of the association between the level of CRP and OS in patients with bone neoplasms stratified by ethnicity (A), histology (B), metastasis (C) and sample size (D). Summary of estimated hazard ratios (HRs) and 95% CI for patients stratified by (A) ethnicity, (B) histology, (C) metastasis and (D) sample size.

To detect the potential source of heterogeneity, the subgroup analyses were stratified by ethnicity, histology, metastasis and sample size (Table 2, Fig 3). As presented in Table 2, the relationship between the level of CRP and OS was not significant in Asian populations (HR = 1.73; 95% CI: 0.86–3.49; P = 0.125) (I2 = 44.4%; P = 0.18). However, the elevated CRP predict poorer OS in patients in Europe (HR = 1.96; 95% CI: 1.51–3.34; P = 0.013) (I2 = 77.4%; P = 0.01). We also performed subgroup analyses on histology, metastasis and sample size to further explain the results of this meta-analysis. Among patients with osteosarcoma, increased CRP was correlated with shortened OS (HR = 1.52; 95% CI: 1.10–2.09; P = 0.01) (I2 = 17.8%; P = 0.30), and the same was true for patients with other kinds of bone cancer (HR = 1.87; 95% CI: 1.28–2.75; P = 0.02) (I2 = 76.0%; P = 0.04). An increased level of CRP was correlated with decreased survival in patients regardless of metastasis (Table 2). Similar trends were also observed when stratified according to sample size (Table 2).

**Table 2:** A summary of HRs for the overall and subgroup analyses of CRP levels in patients with bone neoplasms.

		No. of studies	No. of participants	HR	95%CI	P	I^2^ (%)
**Overall**		5	816	1.87	1.28–2.75	0.001	62.4%
**Ethnicity**	Asian	2	247	1.73	0.86–3.49	0.125	44.4%
	European	3	569	1.96	1.51–3.34	0.013	77.4%
**Histology**	osteosarcoma	3	326	1.52	1.10–2.09	0.01	17.8%
	Osteosarcoma and other kinds	2	490	1.87	1.28–2.75	0.02	76.0%
**Metastasis**	Yes	3	326	1.52	1.10–2.09	0.01	17.8%
	No	2	490	1.87	1.28–2.75	0.02	76.0%
**Sample size**	<100	2	164	1.67	1.02–2.75	0.04	51.1%
	≥100	3	652	1.99	1.06–3.74	0.03	69.6%

### Publication bias and sensitivity analysis

**Fig 4:**
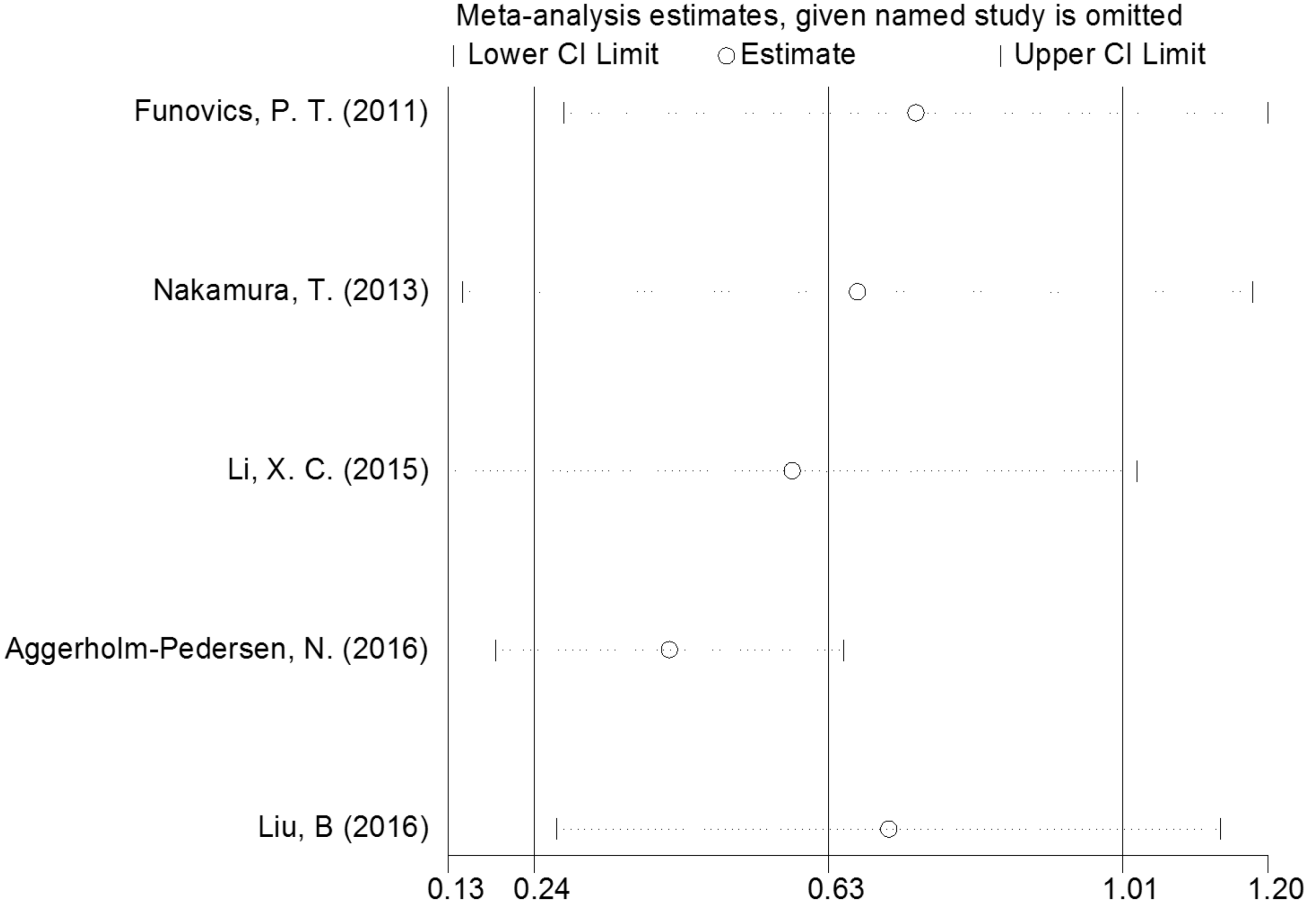
Sensitivity analysis of the relationship between CRP level and OS in bone neoplasms. Sensitivity analyses were performed by excluding each study individually from the meta-analysis.

**Fig 5:**
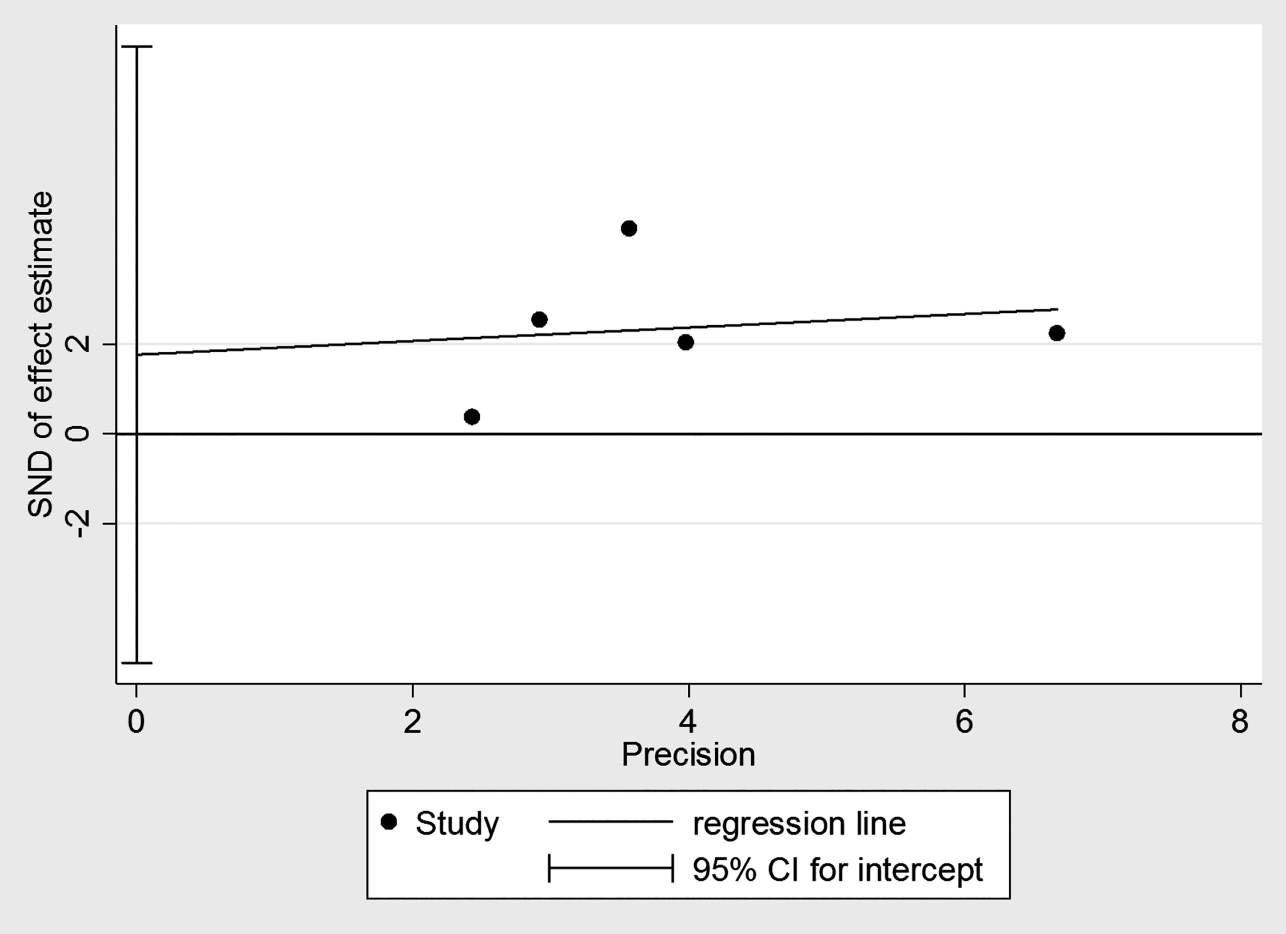
Begg’s funnel plot of the publication bias test for CRP level and OS in bone neoplasms. Summary of funnel plots of publication bias for the included studies. They are funnel plots of the publication bias for this meta-analysis of hazard ratios (HRs).

Significant heterogeneity was discovered among all studies (I^2^ = 62.4%, P = 0.031). The influence of each individual study on the combined HRs was evaluated by systematically deleting one included study at a time. The results showed that the pooled HRs for OS were robust in our study (Fig 4). Moreover, Egger’s test showed no evidence of obvious publication bias (P = 0.473) (Fig 5).

## Discussion

The current meta-analysis summarized the results of five retrospective studies, involving a total of 816 participants. By combining the HRs and 95% CIs from all studies, we showed the association between preoperational serum levels of CRP and the overall survival of patients with bone neoplasms. Our result revealed that higher levels of CRP are associated with shorter OS, with an HR of 1.87 (95% CI: 1.28–2.75; P = 0.001), indicating that high serum levels of CRP before treatment may be a negative prognostic factor for patients with bone cancers. However, stratified analysis by region showed no significant relationship between the level of CRP and OS in Asia (HR = 1.73; 95% CI: 0.86–3.49; P = 0.125). There might be several reasons for this result. First, the susceptibility genes for bone neoplasms in Asia are different from those in Europe, which might lead to different levels of CRP in patients with bone neoplasms. Second, the use of treatment regimens in Asia was significantly different from that in Europe, which might explain the lack of a correlation between the level of CRP and OS in Asia. Finally, the instruments measuring the levels of CRP were not the same in different places, which might cause the different results in Asia and Europe.

Inflammation has been proven to be closely related to all stages of cancer development. It may contribute to cancer initiation by supplying reactive oxygen and nitrogen species that damage DNA directly and it may alter DNA methylation and histone modification, thereby influencing gene expression [23]. Inflammation also facilitates tumor promotion by producing growth factors to sustain proliferation, survival factors to limit cell death, and proangiogenic factors to increase neovascularization [24]. Furthermore, inflammation can assist metastatic progression by providing inductive signals that activate epithelial–mesenchymal transition and extracellular matrix-modifying enzymes that aid tumor invasion, as well as by suppressing anti-tumor immune response [25]. Elevated levels of systemic inflammation have been indicated as being associated with worse survival in patients with solid tumors [26, 27]. Other inflammation biomarkers including the NLR (neutrophil to lymphocyte ratio), the PLR (platelet to lymphocyte ratio) and the mGPS (modified Glasgow prognostic score) could reflect the cancer-related inflammatory status and have been used as prognostic indicators in other cancers [28–32]. As an important biomarker of systemic inflammation, CRP is synthesized by liver cells in response to microbial invasion or tissue injury [33]. CRP is considered a non-specific but sensitive marker of inflammation. While it is well established that CRP levels rise rapidly during acute infection, inflammation, and tissue damage, elevated CRP levels are also seen as an important risk factor for atherosclerosis [34], stroke [35–37], and myocardial infarction [38]. Given that the inflammatory response plays a vital role in cancer, it is not surprising to find increased CRP levels in various cancers. In fact, the serum CRP level before treatment has been proven to be an independent prognostic factor in hepatocellular [39], esophageal [40, 41], renal [42, 43], bladder [44], prostate [45], colorectal [46], ovarian [47], pancreatic [48], and non-small cell lung cancer [49]. Large prospective studies looking for associations between circulating concentrations of CRP and cancer risks have produced conflicting results. Positive relationships were found between serum CRP levels and the increased risk of colorectal and lung cancers, while other studies indicated no relationship between CRP levels and breast, prostate or colorectal cancers [50]. Although the exact functional mechanism of CRP in the development of cancer remains obscure, several hypotheses have been proposed to explain this relationship. First, a causality model has been proposed wherein chronic inflammation causes the elevation of CRP levels, which initiates the formation of malignant tumors. Second, a reverse causality model has been proposed wherein tumor growth and invasion induces tissue inflammation, leading to the increase in CRP levels. A third proposed mechanism states that the body’s innate and adaptive immune systems may react to tumor antigens by increasing CRP levels. A fourth proposed mechanism cites the fact that tumor cells can produce CRP themselves, and they are also able to release cytokines such as IL-6 and IL-8, which contribute to the increase in CRP levels.

The results of this meta-analysis support increased levels of CRP as a prognostic factor for OS in bone cancer, which agrees with the results of most studies [18–20, 22]. We noticed that the previous study carried out by Yi, J. H. also evaluated whether the level of CRP was correlated with the outcome of patients with osteosarcoma [51]. The literature enrolled in that study only included studies published by 2013, and only two studies were included. Unlike that previous study, we had stricter inclusion criteria and enrolled 3 articles published in 2015 and 2016, including a relatively larger number of participants with detailed information. In addition, the research conducted by Yi, J. H. did not evaluate the association between CRP level and OS stratified by ethnicity, histology, metastasis or sample size, as we did. In addition, we concluded that there was no significant relationship between the CRP level and OS in Asian populations based on data from only two studies; therefore, the results should be interpreted with caution. Furthermore, three of the enrolled studies only included patients with osteosarcoma [19–21], although the remaining studies included patients with other kinds of bone cancer [18, 22]. As we know, different kinds of bone cancer might have different overall survival times, which may have contributed to the high heterogeneity in this meta-analysis. An elevated CRP level was defined as less than 10 mg/dl in all studies except one [18], which might also explain the high degree of heterogeneity. All survival data were extracted from multi-factor analyses adjusted for potential confounding factors, including gender, age, stage, treatment and other biomarkers. However, due to the limited information presented in the studies, it was not possible to perform a subgroup analysis according to all cofounding factors.

This research does have several limitations. First, obvious heterogeneity existed in this meta-analysis. Although the sensitivity analysis and the publication bias test indicated the credibility of the results, we could not rule out the cofounding factors or the study criteria that may have resulted in discrepancies between the studies. Second, all the studies included were retrospective studies instead of prospective ones, and therefore the result may represent reverse causation, survival bias or confounding. Third, this study only focused on the CRP level before treatment, and to further investigate its prognostic value, the levels of CRP after surgery and at recurrence should also be taken into consideration.

Generally, our meta-analysis demonstrated the prognostic value of increased preoperative levels of CRP for poorer OS in patients with bone cancer in Europe but not in Asia. However, given the limitations mentioned above, these findings should be treated with caution when applied to clinical practice. More prospective cohort studies are warranted to confirm our results.

## Supporting information

S1 Table Search strategy for meta-analysis of prognostic value of C-reactive protein levels in patients with bone neoplasms. (DOCX)

S1 Checklist PRISMA checklist for meta-analysis of prognostic value of C-reactive protein levels in patients with bone neoplasms. (DOC)
