# Supplementary material for: Prognostic value of C-reactive protein levels in patients with bone neoplasms: A meta-analysis
Source: PLoS One. 2018 Apr 18;13(4):e0195769. doi: 10.1371/journal.pone.0195769 (PMC5906001; doi:10.1371/journal.pone.0195769)
Supplement: S1 Table — (DOCX) [file pone.0195769.s001.docx]

**Table S1. Search strategy for meta-analysis of Prognostic value of C-reactive protein levels in patients with bone neoplasms (PubMed via NLM)**

|  | **Search terms: *C-Reactive Protein and bone neoplasms*** | **Items found** |
| --- | --- | --- |
| ***Population: persons with lung cancer*** | | |
| 1 | (((((((Bone Neoplasm) OR Cancer of Bone) OR Cancer of the Bone) OR Bone Cancer) OR Neoplasms, Bone) OR Neoplasm, Bone)) OR "Bone Neoplasms"[Mesh] | **221573** |
| ***Intervention (Expose): C-Reactive Protein*** | | |
| 2 | (((C Reactive Protein) OR Protein, C-Reactive)) OR "C-Reactive Protein"[Mesh] | **66020** |
| ***Study types*** | | |
| 3 | (((Clinical Trial[Publication Type]) OR review[Publication Type]) OR cohort study[Publication Type]) OR case control study[Publication Type] | **3115034** |
| ***Combined sets*** | | |
| 4 | 1 and 2 and 3 | **294** |
| ***Limits*** | | |
| 5 | 4 AND English[Language] | **294** |

[Mesh] = Term from the Medline controlled vocabulary, including terms found below this term in the Mesh hierarchy
